# Supplementary material for: Changing Exposure Perceptions: A Randomized Controlled Trial of an Intervention with Smoking Parents
Source: Int J Environ Res Public Health. 2020 May 12;17(10):3349. doi: 10.3390/ijerph17103349 (PMC7277098; doi:10.3390/ijerph17103349)
Supplement: Supplementary file 1 [file ijerph-17-03349-s001.pdf]

Supplementary Figure 1:

Parental perceptions of exposure questionnaire (English translation)

A. In this questionnaire you will be presented with various situations showing smokers and children.
To what degree do you think the child in the picture is exposed to cigarette smoke? (To what degree does the smoke reach him/her?).
Rate your answer from 1=not at all to 7=highly

	1	2	3	4	5	6	7	
not at all exposed	O	O	O	O	O	O	O	highly exposed

1. 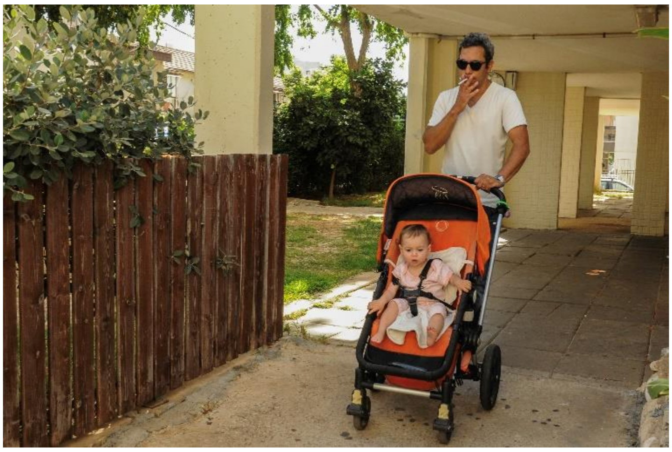

	1	2	3	4	5	6	7	
not at all exposed	O	O	O	O	O	O	O	highly exposed

2.

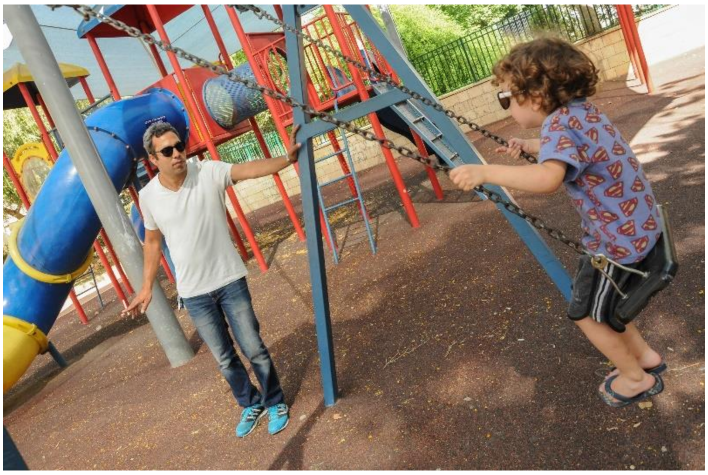

	1	2	3	4	5	6	7	
not at all exposed	O	O	O	O	O	O	O	highly exposed

3. 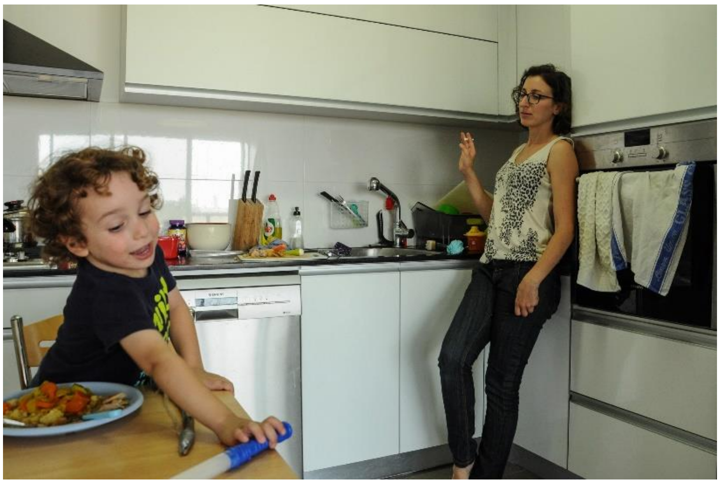

	1	2	3	4	5	6	7	
not at all exposed	O	O	O	O	O	O	O	highly exposed

4. 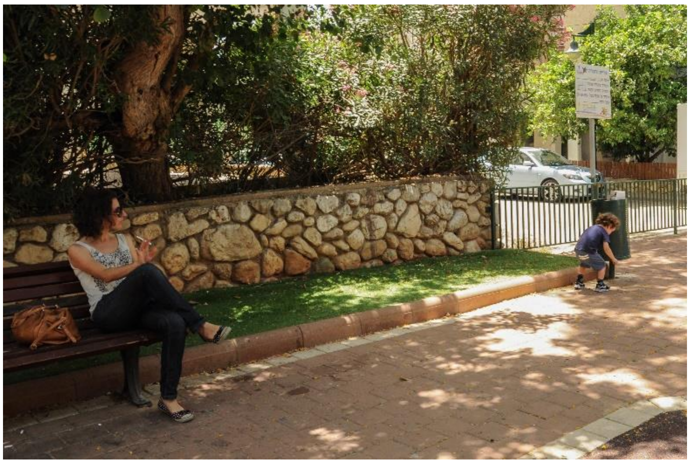

	1	2	3	4	5	6	7	
not at all exposed	○	○	○	○	○	○	○	highly exposed

5. 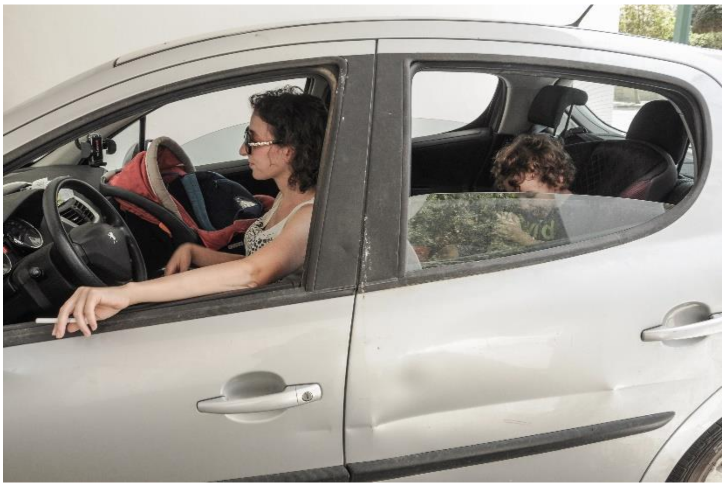

	1	2	3	4	5	6	7	
not at all exposed	○	○	○	○	○	○	○	highly exposed

6. 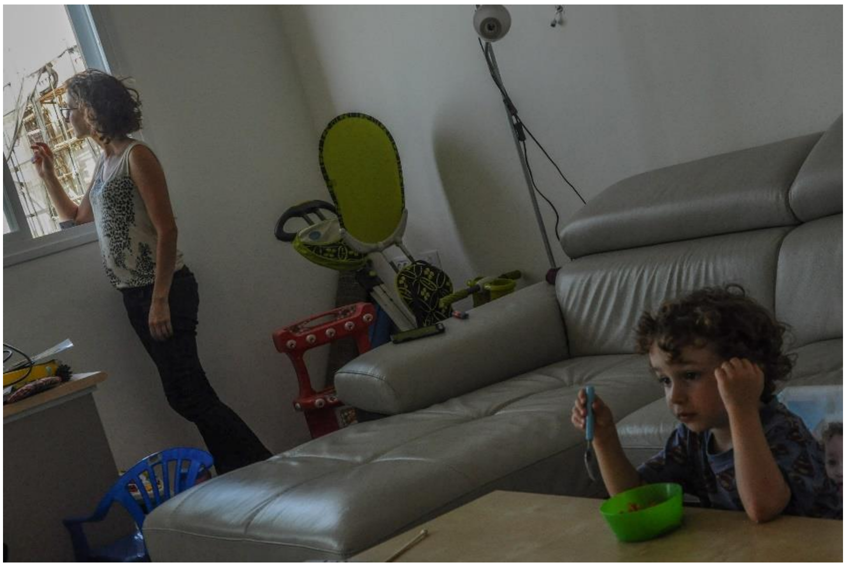

	1	2	3	4	5	6	7	
not at all exposed	O	O	O	O	O	O	O	highly exposed

7. 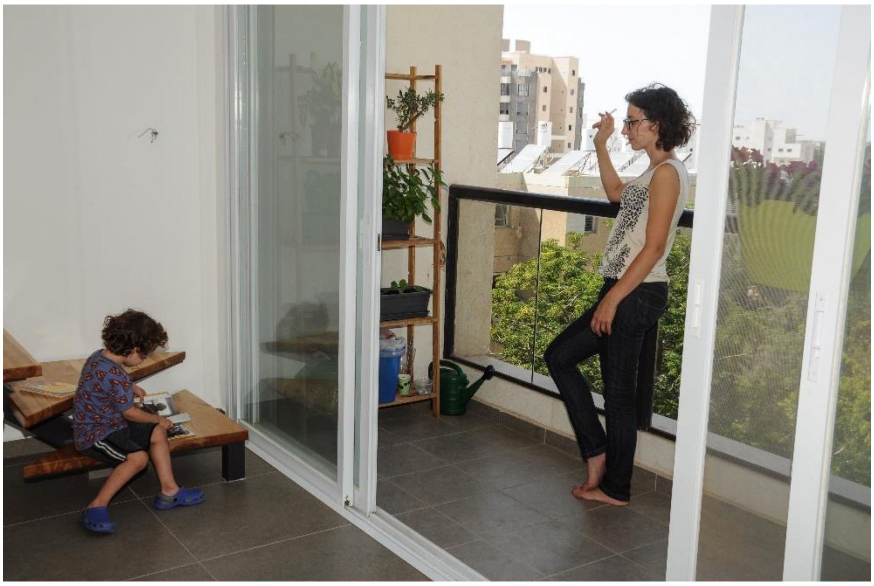

	1	2	3	4	5	6	7	
not at all exposed	O	O	O	O	O	O	O	highly exposed

8. 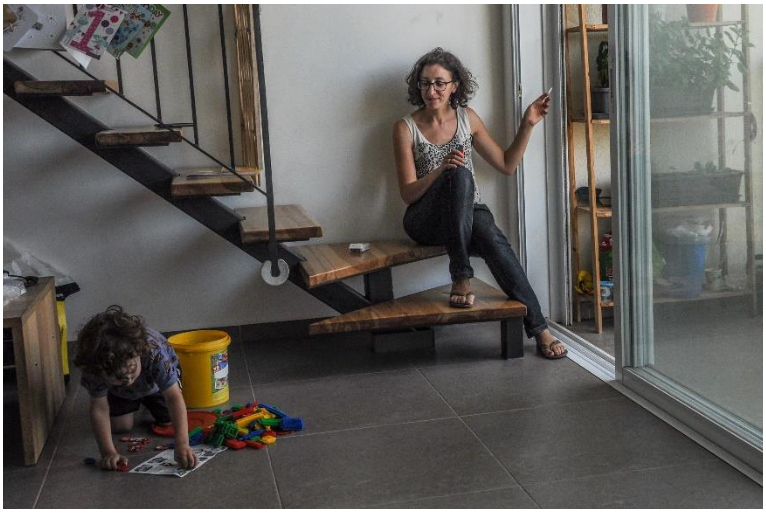

	1	2	3	4	5	6	7	
not at all exposed	O	O	O	O	O	O	O	highly exposed

B. In the following questions situations will be described without pictures. Please rate to what degree you think the child described is exposed to cigarette smoke? (To what degree does the smoke reach him/her?)

9. The child is in the kitchen, someone is smoking on the adjacent service balcony

	1	2	3	4	5	6	7	
not at all exposed	O	O	O	O	O	O	O	highly exposed

10. The child is in a room where someone smoked 12 hours ago

	1	2	3	4	5	6	7	
not at all exposed	O	O	O	O	O	O	O	highly exposed

11. The child is in a room where someone smoked 2 hours ago

	1	2	3	4	5	6	7	
not at all exposed	O	O	O	O	O	O	O	highly exposed

12. The child is in a room where someone smoked 30 minutes ago

	1	2	3	4	5	6	7	
not at all exposed	O	O	O	O	O	O	O	highly exposed

13. The child is in a car where someone smoked 1 hour ago

	1	2	3	4	5	6	7	
not at all exposed	O	O	O	O	O	O	O	highly exposed

14. The child is in a car where someone smoked 20 minutes ago

	1	2	3	4	5	6	7	
not at all exposed	O	O	O	O	O	O	O	highly exposed

15. The child is in the playground and sees his mother smoking but does not smell smoke

	1	2	3	4	5	6	7	
not at all exposed	O	O	O	O	O	O	O	highly exposed

16. The child is in the playground and smells cigarette smoke but does not see the smoker

	1	2	3	4	5	6	7	
not at all exposed	O	O	O	O	O	O	O	highly exposed

17. The child is in the playground and sees his mother smoking and can smell the smoke

	1	2	3	4	5	6	7	
not at all exposed	○	○	○	○	○	○	○	highly exposed

18. What proportion of tobacco smoke is invisible?
    20%
    50%
    80%

19. After smoking in the home, how long does it take for the home to be smoke-free?_____________________________ (open response)

20. After smoking in the car, how long does it take for the car to be free of smoke? _____________________________ (open response)

21. Do you consider yourself to have sufficient information on the subject of passive smoking?

	1	2	3	4	5	6	7	
not at all	○	○	○	○	○	○	○	very much

22. How confident did you feel of your answers?

	1	2	3	4	5	6	7	
not at all	○	○	○	○	○	○	○	very much

23. Did you find it difficult to answer the questionnaire?

	1	2	3	4	5	6	7	
not at all	○	○	○	○	○	○	○	very much

Thank you for your participation!
